# War Demobilisation: Payments for Nurses

**Published:** 1919-02-15

**Authors:** 


### War Demobilisation: Payments for Nurses.

*To the Editor of* The Hospital.

Sir,—I note with interest the very fair and well-thought-out Demobilisation Payment Scheme of the Joint War Committee, which is published in The Hospital of January 25, and should be very grateful if you could tell me whether a similar scheme has been prepared by the War Office for members of the military nursing services, who will also be demobilised very shortly, and many of whom have put in more than four years' service in Territorial and war hospitals. So far, no information has been received by these members as to whether they will receive any post-demobilisation pay.—Yours truly,

Military Nurse.

[In reply to enquiries at the Matron-in-Chief's Office, Territorial Force Nursing Service, we are informed that her department is not aware of any demobilisation scheme drawn up to apply to Territorial Force Nursing Service members who have or may shortly become demobilised. Mr. J. C. Ould, for the Assistant Financial Secretary of the War Office, writes under date of February 8, that a scheme with regard to members of Queen Alexandra's Imperial Military Nursing Service Reserve and Territorial Force Nursing Service is at present under consideration, and it is therefore regretted that it is not possible to give particulars of it. Mr. Ould continues: "I am to add that no such scheme is necessary with regard to members of Queen Alexandra's Imperial Military Nursing Service, as these are permanent employees of the War Department and will not be demobilised."—Ed. The Hospital.]

